# Supplementary material for: Empowering adult patients with diabetes for health educators’ role within their family members: A cross-sectional study
Source: PLoS One. 2024 Apr 16;19(4):e0299790. doi: 10.1371/journal.pone.0299790 (PMC11020498; doi:10.1371/journal.pone.0299790)
Supplement: S1 File — (PDF) [file pone.0299790.s002.PDF]

King Abdullah International Medical Research Center
KAIMRC
APPROVED
IRB
Institutional Review Board

NRC22R/338/08

الاستبانة

**معلومات شخصية عامة**	
1 . ما هو عمرك؟	
2 . ما هو جنسك؟	1. ذكر<br>2. أنثى
3 . ما هي حالتك الاجتماعية؟	1. غير متزوج<br>2. متزوج<br>3. أرمل<br>4. مطلق
4 . هل لديك أبناء/بنات؟	1 . نعم<br>2 . لا
5 . إذا كان الجواب نعم، ما هو عدد أبناءك وبناتك؟	
6 . ما حجم عائلتك؟	1 . عائلة الصغيرة: شخصان بالغان مع طفل واحد على الأقل.<br>2 . عائلة ذات والد وحيد: شخص بالغ واحد فقط يقوم بتربية الأطفال.<br>3 . عائلة ممتدة: أحد الوالدين أو الوالدين، والأطفال، والأجداد، والعمات، والأعمام، او أبناء العم يعيشون معًا.<br>4 . عائلة مختلطة: وجود زوج الام/ زوج الاب مع العائلة.
7 . ما هو دورك داخل عائلتك؟	1 . ابن<br>2 . بنت<br>3 . الأم<br>4 . أب<br>5 . جدة<br>6 . جد
8 . ما هو وضعك الوظيفي؟	1 . دوام كامل<br>2 . اعمل بدوام جزئي<br>3 . عاطل عن العمل<br>4 . متقاعد<br>5 . طالب
9 . ما هو الدخل الشهري لعائلتك؟	1 . 5000ريال او اقل<br>2 . 5000-10000 ريال<br>3 . 10000-15000 ريال<br>4 . 15000 –20000 ريال<br>5 . 20000ريال او اكثر
10 . ما هو مستواك التعليمي؟	1 . غير متعلم<br>2 . شهادة الثانوية العامة<br>3 . دبلوم عالي<br>4 . درجة بكالوريوس<br>5 . درجة الماجستير<br>6 . درجة الدكتوراه
**معلومات متعلقة بمرض السكري**	

King Abdullah International Medical Research Center
KAIMRC
APPROVED
IRB
Institutional Review Board

1. هل لديك تاريخ عائلي للإصابة بمرض السكري من النوع 2 لدى أقارب من الدرجة الأولى؟	1. نعم<br>2. لا
2. هل لديك تاريخ عائلي لمرض السكري من النوع 2 لدى أقارب من الدرجة الثانية؟	1. نعم<br>2. لا
3. منذ كم سنة تم تشخيصك ب مرض السكري؟	1. سنه او اقل<br>2. سنه الي ٥ سنوات<br>3. من ٦ الي ١٠ سنوات<br>4. من ١١ سنه الي ١٦ سنه<br>5. ١٧ سنة فما فوق
4. ما هي خطتك العلاجية؟	1. لا توجد أدوية فقط حمية غذائية<br>2. حبوب لعلاج السكري<br>3. الأنسولين<br>4. كلاهما
أي من المشاكل التالية المرتبطة أحيانًا بمرض السكري، إن وجدت، قد عانيت منها؟	
1. انخفاض سكر الدم (<80 مجم / ديسيلتر)	1. نعم<br>2. لا
2. ارتفاع نسبة السكر في الدم (> 300 مجم / ديسيلتر)	1. نعم<br>2. لا
3. مشاكل قلبية	1. نعم<br>2. لا
4. مشاكل في الكلى	1. نعم<br>2. لا
5. الصعوبات الجنسية	1. نعم<br>2. لا
6. تلف شبكية العين	1. نعم<br>2. لا
7. تلف الأعصاب (على سبيل المثال، تنميل أو وخز في اليدين أو القدمين أو تقرحات القدم)	1. نعم<br>2. لا
8. هل لديك أمراض في القلب والأوعية الدموية	1. نعم<br>2. لا
5. هل سبق لك أن تلقيت توعية بشأن مرض السكري من طبيب / اختصاصي تغذية / ممرضة في الماضي؟	1. نعم<br>2. لا

King Abdullah International Medical Research Center
KAIMRC
APPROVED
IRB
Institutional Review Board

العوائق والميسرات ومدى استعداديتك لتكون مثقف صحي لمجتمعك

الرجاء تصنيف موافقتك على العبارات التالية:

العبارة	موافق بشدة	موافق	محايد	لا موافق	لا أوافق بشدة
1. مرض السكري هو مرض خطير يمكن أن يضر بالصحة.					
2. أفراد عائلتي وأقاربي معرضون لخطر الإصابة بمرض السكري.					
3. إذا قمت بتثقيف أفراد عائلتي وأقاربي حول مرض السكري، فسيكونون أكثر وعياً بأهمية تغيير نمط الحياة للوقاية من مرض السكري.					
4. إذا قمت بتثقيف أفراد عائلتي وأقاربي حول مرض السكري، فسيتم تشجيعهم على إجراء تغييرات في نمط الحياة.					
5. إذا قمت بتثقيف أفراد عائلتي وأقاربي حول مرض السكري، فسيكون لديهم خطر أقل للإصابة بمرض السكري.					
6. أنا على دراية بالوقاية من مرض السكري.					
7. أعرف كيف أبلغ أفراد عائلتي بالوقاية من مرض السكري.					
8. أعرف أفراد الأسرة أو الأقارب الذين يجب تثقيفهم حول مرض السكري.					
9. لقد تحدثت بالسابق مع عائلتي حول استراتيجيات الوقاية من مرض السكري.					
10. أنا على استعداد للعمل كمثقف صحي لأفراد عائلتي وأقاربي المقربين.					
11. لدي القدرة على العمل كمثقف صحي عن الوقاية من مرض السكري لأفراد عائلتي وأقاربي المقربين.					
12. تعليمي وتهيئتي في التثقيف الصحي عن الوقاية من مرض السكري سيزيد من ثقتي بنفسي كمثقف صحي.					
13. يجب أن أتلقى تثقيفًا صحيًا قبل أن أقوم بتعليم أفراد عائلتي وأقاربي المقربين عن الوقاية من مرض السكري.					
14. أنا قلق بشأن إصابة أفراد عائلتي وأقاربي بمرض السكري.					
