# Supplementary material for: Empowering adult patients with diabetes for health educators’ role within their family members: A cross-sectional study
Source: PLoS One. 2024 Apr 16;19(4):e0299790. doi: 10.1371/journal.pone.0299790 (PMC11020498; doi:10.1371/journal.pone.0299790)
Supplement: S2 File — (PDF) [file pone.0299790.s003.PDF]

NRC22R/338/08

King Abdullah International Medical Research Center
KAIMRC
APPROVED
IRB
Institutional Review Board

Measurements

Socio-demographics	
1. What is your age?	
2. What is your gender?	Male<br>Female
3. What is your marital status?	1) Never married<br>2) Married<br>3) Widowed<br>4) Divorced
4. Do you have offspring?	Yes/ No
5. If yes, what is your number of offspring?	
6. What is your family type?	a. Nuclear family,<br>b. single parent,<br>c. extended family,<br>d. blended Family.
7. What is your role within your family?	a. Son<br>b. Daughter<br>c. Mother<br>d. Father<br>e. Grandmother<br>f. Grandfather
8. What is your employment status?	a. Employed full-time<br>b. Employed part-time<br>c. Unemployed<br>d. Retired<br>e. Student
9. What is your family monthly income?	a. < 5000RS<br>b. 5000-10000RS<br>c. 10000-15000RS<br>d. 15000- 20000RS<br>e. >20000RS
10. What is your educational level?	a. Uneducated<br>b. High school certificate<br>c. Higher Diploma<br>d. Bachelor’s degree<br>e. Master’s degree<br>f. Doctorate (PhD)
**Diabetes-related background**	

King Abdullah International Medical Research Center
KAIMRC
APPROVED
IRB
Institutional Review Board

1. Do you have a family history of T2D in first degree relatives?	Yes/ No
2. Do you have a family history of T2D in second degree relatives?	Yes/ No
1. How many years have you lived with diabetes?	a. Less than 1 years<br>b. 1- 5 years<br>c. 6-10 years<br>d. 11-16 years<br>e. 17 years and above
2. What is your treatment plan?	a. No medications just diet<br>b. Tablets<br>c. Insulin<br>d. Both
3. Which if any, of the following problems sometimes associated with diabetes have you experienced?	a. Low blood sugar (<80mg/dl)<br>b. High blood sugars (>300 mg/dl)<br>c. Heart problems<br>d. Kidney problems<br>e. Sexual difficulties<br>f. Damage to the retina of the eye<br>g. Nerve damage (e.g., numbness or tingling of the hands or feet, or foot ulcers)<br>h. None of the above
4. Do you have any other comorbidity?	Cardiovascular problems
5. Have you ever received diabetes education from physician/dietician/nurse in the past?	Yes/ No

Facilitators, Barriers, Willingness, and Readiness

Rate your agreement on the following statements:					
Statement	Strongly agree	Agree	Natural	Disagree	Strongly disagree
1. Diabetes is a serious disease that can cause harm to health.					
2. My family members and close relatives are at risk for develop diabetes.					
3. If I educate my family members and close relatives about diabetes, they will be more aware of the importance of lifestyle changes to prevent diabetes.					
4. If I educate my family members and close relatives about diabetes, they will be encouraged to make lifestyle changes.					

King Abdullah International Medical Research Center
KAIMRC
APPROVED
IRB
Institutional Review Board

5. If I educate my family members and close relatives about diabetes, they will have less risk of developing diabetes.					
6. I am knowledgeable about diabetes prevention					
7. I know how to inform my family members about diabetes prevention.					
8. I know which family members or close relative to educate about diabetes.					
9. I already have talked to my family about strategies to prevent diabetes.					
10. I am willing to act as a health educator for my family members and close relatives.					
11. I have the ability to act as a health educator for my family members and close relatives.					
12. Health education preparation will increase my confidence as health educator.					
13. I must receive professional health education before I educate my family members and close relatives.					
14. I am worried about my family members and close relative developing diabetes.					
