# Supplementary material for: Empowering adult patients with diabetes for health educators’ role within their family members: A cross-sectional study
Source: PLoS One. 2024 Apr 16;19(4):e0299790. doi: 10.1371/journal.pone.0299790 (PMC11020498; doi:10.1371/journal.pone.0299790)
Supplement: Attachment — Submitted filename: RESPON~2.DOC [file pone.0299790.s005.DOC]

PONE-D-23-32756

Empowering Patients with Diabetes for Health Educators' Role within Their Family Members: A Cross-sectional Study

PLOS ONE


Please include the following items when submitting your revised manuscript:

Journal Requirements:

When submitting your revision, we need you to address these additional requirements.

1. Please ensure that your manuscript meets PLOS ONE's style requirements, including those for file naming. The PLOS ONE style templates can be found at
   <https://journals.plos.org/plosone/s/file?id=wjVg/PLOSOne_formatting_sample_main_body.pdf> and 
   <https://journals.plos.org/plosone/s/file?id=ba62/PLOSOne_formatting_sample_title_authors_affiliations.pdf> Dr. Mona- DONE as requested.

2. We note that you have indicated that data from this study are available upon request. PLOS only allows data to be available upon request if there are legal or ethical restrictions on sharing data publicly. For more information on unacceptable data access restrictions, please see <http://journals.plos.org/plosone/s/data-availability#loc-unacceptable-data-access-restrictions>.

In your revised cover letter, please address the following prompts:

a) If there are ethical or legal restrictions on sharing a de-identified data set, please explain them in detail (e.g., data contain potentially sensitive information, data are owned by a third-party organization, etc.) and who has imposed them (e.g., an ethics committee). Please also provide contact information for a data access committee, ethics committee, or other institutional body to which data requests may be sent.

b) If there are no restrictions, please upload the minimal anonymized data set necessary to replicate your study findings as either Supporting Information files or to a stable, public repository and provide us with the relevant URLs, DOIs, or accession numbers. For a list of acceptable repositories, please see <http://journals.plos.org/plosone/s/data-availability#loc-recommended-repositories>. Dr. Mona- DONE as requested.

We will update your Data Availability statement on your behalf to reflect the information you provide.

3. Your ethics statement should only appear in the Methods section of your manuscript. If your ethics statement is written in any section besides the Methods, please move it to the Methods section and delete it from any other section. Please ensure that your ethics statement is included in your manuscript, as the ethics statement entered into the online submission form will not be published alongside your manuscript.  Dr. Mona- DONE as requested.

4. Please review your reference list to ensure that it is complete and correct. If you have cited papers that have been retracted, please include the rationale for doing so in the manuscript text, or remove these references and replace them with relevant current references. Any changes to the reference list should be mentioned in the rebuttal letter that accompanies your revised manuscript. If you need to cite a retracted article, indicate the article’s retracted status in the References list and also include a citation and full reference for the retraction notice. Dr. Mona- DONE as requested.

Additional Editor Comments :

Thank you for the submitting this manuscript to PLOS ONE. This study has a significant impact on diabetes prevention measures. However, I have several important concerns

♣ Page 11-line 72-73: The author hypothesized that diabetes prevention programs would reduce healthcare system costs; however, this study did not examine the cost effectiveness of involving patients in the education program, consider rewrite it please. Dr. Mona- DONE as requested.

1. This study was carried out on diabetic adults. This should be reflected in the title and the abstract (page 2, line 39). Dr. Mona- DONE as requested.
2. Were there Saudis and non-Saudis among the participants? Clarifying this point will be useful for future research. Dr. Mona- DONE as requested, under the setting and participants’ section.
3. Pilot testing was conducted among 10% of the total required sample size. Were those patients included in the analysis? Dr. Mona - No they were not, DONE as requested, in the Reliability and Validity
4. Page 13, line 266: the Cronbach's alpha coefficient of Perceived Benefits and Perceived Barriers was 0.77; however, table 3 reported a different value (0.79). Dr. Mona- DONE as requested.
5. Page 13, line 268: The Cronbach's alpha coefficient of the self-efficacy scale was 0.78, but table three reported a different value. Please correct. Dr. Mona- DONE as requested.
6. Page 12, line 240: The author reported the difference in self-efficacy, but the data was not presented or provided (one-way analysis of variance (ANOVA) and Tukey’s HSD post-hoc tests). Response: Dr. Mona- Regarding this point as reported in the results section with running the ANOVA and Tukey’s HSD post-hoc tests, the results reported in the paragraph with significant difference, for total scale score and self-efficacy reporting F and p value (ANOVA), then reporting the difference in means score and standard deviation for also the total scale mean score and self-efficacy (Tukey’s HSD post-hoc tests).
7. Page 8, line 148: What is the cute off point that is considered a lower score? (i.e., indicates the need for interventions). Dr. Mona- DONE as requested, in the same paragraph.
8. Result: Table 1, the many significant findings were reported in table 3, the authors did not investigate and explained such as:
   - Perceived benefits/barriers vs Marital Status /family income
   - Perceived benefits/barriers vs family income
   - Cues to Action (worries level) vs Education level/age.
   - Perceived susceptibility vs Employment Status and education level. Response: Dr. Mona – thank you for highlighting this. All of the mentioned items were added to the results and discussion sections as requested.
9. The author report “Participant’s willingness to be a health educator” as separate binary question, however, this question displayed in the survey tool as 5-point Likert scale.?? Response: Dr. Mona- Yes, we recalculated the responses for this question, categorizing the answers into 'Yes' or 'No'. Specifically, those who responded with 'Strongly agree' and 'Agree' were considered as 'Yes', while those who responded with 'Neutral', 'Disagree', or 'Strongly disagree' were categorized as 'No'. this part is now added to the Statistical Analysis section.
10. “Vancouver” style should be used as per PLOS On submission guideline. Dr. Mona- DONE as requested.

5. Review Comments to the Author

Please use the space provided to explain your answers to the questions above. You may also include additional comments for the author, including concerns about dual publication, research ethics, or publication ethics. (Please upload your review as an attachment if it exceeds 20,000 characters)

Reviewer #1: I would alter language- instead of diabetic patient, please use people living with diabetes(always) especially as you are promoting a person centered approach
The subject is very topical and serves as an important opportunity to engage service users as educators. It is more likely that advice would be adhered to if delivered by peers and more so if family members.
I am glad it was pointed out that structured education prior to enlisting candidates was important.
Testing their knowledge and understanding would need to be considered in the next iteration.
Results of this approach such as hba1c etc would be valuable to assess the effectiveness of this approach. Response: thank you so much for your valuable feedback, your comments were taken into consideration.

Reviewer #2: Thank you for providing opportunity to review the manuscript, “Empowering Patients with Diabetes for Health Educators' Role within Their Family Members: A Cross-sectional Study”, which may have potential impact for the diabetes self-management and care. However, there are some suggestions from my side to make it better.

Abstract
Line 42.
“Various statistical analyses …….”
If authors revise this sentence and be specific that would be great. Dr. Mona- DONE as requested in the abstract

Introduction

Page 4, line 72 to 79.
This paragraph is unclear, initially, authors described the hypothesis regarding the benefits i.e., costs reduction. Then, feasibility etc., which is not connected to the previous paragraph too. This is because in the last sentence of the previous paragraph, you wrote about sense of empowerment, solidarity and shared responsibility.
Therefore, please revised this whole paragraph. Describe how it empower…? You can add cost reduction as an example “For example, previous study by …. Showed that cost was reduced by …. And don’t forget to give the references.

And finally make a connection with your study site… Why is this needed? Dr. Mona- DONE as requested, the whole paragraph was revised as recommended.

Page 9, line 171, Replace “will be asked” by “asked” and more grammatical correction is needed throughout the manuscript. Dr. Mona- DONE as requested

Page 11, line 210.
Could author describe in details of using all statistical analyses? For example, why ANOVA, for which variables?
Response: Dr. Mona, Thank you for this comment, this part was revised in the same paragraph of statistical analysis

Discussion
Discussion section required major revision. Authors should compare and contrast the findings with the previous studies conducted, which is missing in each and every finding.

Such as in page 14, line 284, your findings says that 87% participants were willing to server as a health educator. Are there any previous studies conducted related to these findings? Are the findings similar to your study? OR is it difference? Give reason… what will these findings indicate? Please reorganise your writing in this way. Please follow same style for other major findings such as self-efficacy…
Response: Dr. Mona- thank you so much for raising this and we really understand your point of view, we did revise all of the discussion section with addressing all of your points, however, it's important to highlight that the approach we're studying is groundbreaking and has not been previously explored in the literature. As far as our knowledge extends, this study represents the initial effort to investigate the empowerment of patients as health educators within their family context. Consequently, we've encountered challenges in finding comparative studies to contextualize or benchmark our findings against. However, we tried as much as possible and we added more citations.

In result section, you mentioned significant difference in self-efficacy among the patient’s education and income level. You need to discuss these findings here, why is this difference in you study site? Is there any study to support this? Response: Dr. Mona- thank you so much for your comment. This part was added to the discussion as recommended.

Conclusions
Can you conclude based on you study aim and put these all recommendations at the end.

Are patients willing to serve as a health educator? What are the influencing factors? Response: Dr. Mona- thank you so much for you valued feedback, your comments were taken into consideration, the whole conclusion were revised as requested.

Thank you!!!
